# Transforming growth factor β1 accelerates and enhances in vitro red blood cell formation from hematopoietic stem cells by stimulating mitophagy

**DOI:** 10.1186/s13287-020-01603-z

**Published:** 2020-02-19

**Authors:** Rutuja Kuhikar, Nikhat Khan, Joseph Philip, Sameer Melinkeri, Vaijayanti Kale, Lalita Limaye

**Affiliations:** 1 National Centre for Cell Science, NCCS Complex, Savitribai Phule Pune University Campus, Ganeshkhind, Pune, Maharashtra 411007 India; 2 Armed Forces Medical College, Pune, 411040 India; 3 Blood and Marrow Transplant Unit, Deenanath Mangeshkar Hospital, Erandawne, Pune, 411004 India; 4 Symbiosis Centre for Stem Cell research, School of Biological Sciences, Symbiosis International (Deemed University), Lavale, Pune, 412115 India

**Keywords:** Red blood cells, Apheresis-derived peripheral blood, Hematopoietic stem cells, TGF-β1, Mitophagy

## Abstract

**Background:**

Generation of red blood cells (RBCs) from hematopoietic stem cells (HSCs) in vitro takes about 21 days, making it unaffordable for clinical applications. Acceleration of the in vitro erythropoiesis process by using small molecules could eventually make the large-scale production of these cells commercially viable. Transforming Growth Factor β1 (TGF-β1) has been shown to have a dose-dependent activity on the HSCs: at high concentration it inhibits, whereas at low concentration it stimulates the HSCs growth. At high concentration, it also inhibits erythropoiesis but accelerates terminal erythroid differentiation of cell lines and erythroid progenitors. Here we examined whether the use of low concentration of TGF-β1 would be beneficial for increasing RBC production by stimulating HSC growth and also supporting erythroid differentiation. Such a strategy could make RBC production in vitro more efficient and cost-effective for clinical applications.

**Methods:**

HSCs isolated from Apheresis samples were differentiated into mature RBCs by the sequential addition of specific combinations of growth factors for 21 days. In the control set, only EPO (3 IU/ml) was added whereas, in the test set, TGF-β1 at a concentration of 10 pg/ml was added along with EPO (3 IU/ml) from day 0.

**Results:**

We found that a low concentration of TGF-β1 has no inhibitory effect on the proliferation of the early stages of erythropoiesis. Additionally, it significantly accelerates terminal stages of erythroid differentiation by promoting BNIP3L/NIX-mediated mitophagy.

**Conclusions:**

Incorporation of TGF-β1 at 10 pg/ml concentration in the differentiation medium accelerates the in vitro erythropoiesis process by 3 days. This finding could have potential applications in transfusion medicine.

**Electronic supplementary material:**

The online version of this article (10.1186/s13287-020-01603-z) contains supplementary material, which is available to authorized users.

## Background

Generation of red blood cells (RBCs) from hematopoietic stem cells (HSCs) in vitro take more than 3 weeks, making it cost-ineffective. Considering its importance and eventual application in transfusion medicine, efforts are being made to produce RBCs on a large scale by using feeder layers or suspension cultures [1–7]. However, in spite of these efforts in vitro generation of RBCs from HSCs continues to take about 21 days [1, 2, 5, 6, 8]. Acceleration of this process may make the large-scale production of RBCs affordable by reducing the high cost associated with growth factors, media, etc. required for the culture. Although some reports have documented the use of various molecules for an acceleration of the process, further research is still needed in this field [9–12].

Transforming growth factor β1 (TGF-β1) has been shown to have bidirectional effects on different cell types, including HSCs, wherein it acts as an inhibitor at high concentration and promotes proliferation at low concentration [13–15]. In the case of erythroid differentiation, TGF-β1 at a high concentration was shown to inhibit proliferation and accelerate the terminal erythroid differentiation of erythroleukemia cell lines and erythroid progenitors [10, 11]. However, in these reports, the percent enucleation obtained was less. Kale and Vaidya have shown a stimulatory effect of low TGF-β1 concentration on the HSCs [16, 17]. This prompted us to investigate the effect of low TGF-β1 concentration on the generation of RBCs from HSCs.

Terminal erythroid differentiation involves depletion of intracellular organelles and extrusion of the nucleus from erythroblasts to form fully functional mature RBCs. Autophagy plays a critical role in eliminating organelles during erythroid differentiation [18–20]. In particular, clearance of mitochondria—termed as mitophagy—is an essential process for the final RBC maturation [21–24]. TGF-β1 is known to accelerate terminal erythroid differentiation by arresting or delaying cells in the G1 phase [11]. Role of TGF-β1 in activating autophagy has recently been shown in normal bovine mammary epithelial cells, hepatocellular carcinoma cells, and mammary carcinoma cells [25, 26]. However, its role in activating autophagy, specifically mitophagy, during the in vitro erythropoiesis process has not been elucidated.

Here we show that a low concentration of TGF-β1 does not adversely affect the proliferation of early stages of erythropoiesis. Moreover, the addition of TGF-β1 from the initiation of culture significantly accelerates and enhances the generation of RBCs from the HSCs. Importantly, we also succeeded in obtaining a higher percent of enucleated RBCs at earlier time points compared with previous reports. Mechanistically, we show that TGF-β1 acts by stimulating mitophagy in the late stages of erythroid differentiation.

## Materials and methods

### Ethical approvals for primary human samples

Approvals for the use of a primary human sample (apheresis) were obtained from local hospitals after informed consent with the compliance of the institutional review board (IEC-Institutional ethical committee–NCCS and IC-SCR–Institutional Committee for Stem Cell Research, NCCS) according to the Declaration of Helsinki. Consenting procedures were also approved by the NCCS-ICSCR.

### Collection of apheresis samples

#### Apheresis samples

G-CSF-mobilized peripheral blood, i.e., apheresis samples (APBL) from healthy donors, were collected in sterile heparinized bottles, after obtaining informed consent and processed within 24 h of collection. These were residual samples leftover in the tubings after transplant, and thus, the donor’s safety was not compromised. Hematopoietic stem cells (HSCs) were isolated by the positive selection method using Dynabeads CD34-positive magnetic beads (Invitrogen, Waltham, Massachusetts, USA) according to the manufacturer’s instructions.

#### Cell line

TF1 cell line, a human bone marrow-derived erythroleukemic cell line (ATCC® CRL-2003™), was grown in RPMI-1640 (Sigma-Aldrich, St. Louis MO, USA) containing 10% fetal bovine serum (GIBCO, Grand Island, NY, USA) and 8% Giant Cell Tumor Conditioned Medium (GCT-CM) at 37 °C in humidified 5% CO_2_ atmosphere.

#### In vitro generation of red blood cells (RBCs) from HSCs

HSCs were seeded in 24-well plates at a seeding density of 5 × 10^4^/ml/well in serum-free Iscove’s modified Dulbecco’s medium (IMDM) (Sigma-Aldrich, St. Louis MO, USA), supplemented with 3% human AB^+ve^ plasma, 2% human AB^+ve^ serum, 200 μg/ml iron-saturated human transferrin, 90 ng/ml ferric nitrate, 900 ng/ml ferrous sulfate, and 10 μg/ml insulin (Sigma Aldrich, St. Louis MO, USA).

A three-step protocol was designed for the expansion and generation of RBCs. In the first step (day0–6), HSCs were supplemented with 10^− 6^ M hydrocortisone, 100 ng/ml SCF, 5 ng/ml IL-3 (Peprotech Inc., Rocky Hill, NJ, USA), and 3 IU/ml EPO (Genova Biopharmaceuticals, Pune). The second step (day 7–13) included supplementation of the medium only with EPO (3 IU/ml). In the third step (days 14–21), the medium was additionally supplemented with adenine (0.14 mg/ml), D-mannitol (14.57 mg/ml), and disodium hydrogen phosphate (0.94 mg/ml), and only EPO (3 IU/ml) was added. In the control set, only EPO (3 IU/ml) was added whereas, in the test set, TGF-β1 at a concentration of 10 pg/ml was added along with EPO (3 IU/ml) from day 0. Half feeding was done on day 4 while the media was completely replaced on days 7, 11, 14, and 17.

For the TGF-β1 inhibitor study, SB-431542 (Sigma Aldrich, St. Louis MO, USA) was added at 1 μM from day 7 till day 21.

#### Cell viability assays

TF1 cells were seeded in 96-well plates and treated with different concentrations of TGF-β1 (Peprotech Inc., Rocky Hill, NJ, USA). After 24 and 48 h, cell viability was measured by MTT (3-(4, 5-dimethylthiazol-2-yl)-2,5-diphenyltetrazolium bromide) (Sigma Aldrich, St. Louis MO, USA) assay. Cells were incubated with MTT (10 μl of 5 mg/ml MTT solution per well) for 4 h at 37 °C. The reaction was stopped with the addition of 10% sodium dodecyl sulfate (SDS) (Sigma Aldrich, St. Louis MO, USA). Absorbance values were determined at 570 nm using Multiskan™ FC Microplate Photometer (Thermo Fisher Scientific, Waltham, Massachusetts, USA), and results were expressed as a percentage of control cells without TGF-β1. Cell viability was assessed by the Trypan blue exclusion method at different time points.

Viability of the cultured cells was also determined by Calcein Am staining. Cells were stained with Calcein Am (10 μM) for 30 min at 37 °C in the dark. After incubation,, the cells were washed to remove the excess dye, re-suspended in PBS, and acquired on FACS Canto II (BD, San Jose, CA, USA).

#### Morphologic and phenotypic analysis

Cultured cells were harvested at different time intervals. For morphological characterization, cytospin smears were prepared and stained with Wright’s and Giemsa stain. A total of 500 cells were counted in random non-overlapping fields to determine the different stages of erythropoiesis process.

Phenotypic characterization was done by analyzing the expression of different cell surface markers. Cells were harvested and suspended in 50 μl of 1× phosphate-buffered saline (PBS) containing 0.1% bovine serum albumin (Sigma Aldrich, St. Louis MO, USA) and 0.1% azide (Sigma Aldrich, St. Louis MO, USA), and then incubated with specific fluorochrome-tagged antibodies for 45 min at 4 °C in the dark. Proper isotype-matched antibodies were used as controls. The stained cells were washed, re-suspended in PBS, and acquired on FACS Canto II (BD, San Jose, CA, USA). Data were analyzed by using BD FACS DIVA or BD Flow Jo software. The details of the antibodies used are as follows: CD71-FITC, CD235a-APC (ebioscience, San Diego, California, USA), CD235a-Brilliant violet, CD49d-APC, AnnexinV FITC (BD Bioscience, San Jose, CA, USA).

#### Quantitation of percent enucleation of RBCs

Cells were stained with nucleic acid binding dye SYTO16 (100 nM) for 15 min in the dark. Stained cells were acquired on flow cytometry for assessment of enucleated RBCs by using a combination of SYTO16 fluorescence and forward side scatter (FSC). Enucleated RBC percent is calculated as the percent of SYTO16^−^ cells in the total cells excluding debris and nuclei.

#### Standard hematological variables of RBCs

Culture generated RBCs were assessed for standard hematological parameters such as mean corpuscular volume (MCV), mean cell hemoglobin (MCH), and mean corpuscular hemoglobin concentration (MCHC). These were determined by using an automated hematology analyzer (Sysmex, Kobe, Japan).

The proportion of adult and fetal hemoglobin present in generated RBCs was determined by high-performance liquid chromatography (HPLC) (Bio-Rad, California, USA).

#### Glucose-6-phosphate dehydrogenase activity

Glucose-6-phosphate dehydrogenase (G6PD) activity was determined by the measurement of the rate of increase in NADPH absorbance at 340 nm using a spectrophotometer.

#### Cell cycle

Cultured cells were harvested and washed with 1× PBS. The cells were then fixed with 70% ethanol for 10 min. Further, cells were washed, re-suspended in PBS containing RNAase (Sigma Aldrich, St. Louis MO, USA) (100 μg/ml), and incubated for 30 min at 37 °C. Thereafter, propidium iodide (50 μg/ml) was added and the cells were immediately acquired on FACS Calibur (BD, San Jose, CA, USA).

#### Mitochondrial assay

MitoTracker Green (MTG) (Invitrogen, Waltham, Massachusetts, USA) was used to track mitochondrial mass. Mitochondrial membrane potential was analyzed by tetramethylrhodamine, ethyl esterdye (TMRE) (Invitrogen, Waltham, MA, USA). MitoSOX (Invitrogen, Waltham, MA, USA) was used to determine mitochondrial ROS. Cells were stained individually with MTG (20 nM), TMRE (100 nM), and MitoSOX (2 μM) for 30 min at 37 °C. After staining, cells were washed to remove excess dye and fluorescence intensities were measured by flow cytometry.

#### Quantitative RT-PCR

The mRNA was isolated by the TRIzol method. Isolated mRNAs were reverse transcribed to cDNA by using MMLV reverse transcriptase (Sigma Aldrich). Quantitative real-time PCRs were performed using Platinum® SYBR® Green (DSS Takara), and the data were analyzed on ABI 7500 (Applied Biosystems). GAPDH expression was used to normalize the gene expression of samples. Relative gene expression was calculated as RQ = 2^−ΔΔCt^. Primer sequences (IDT) used are listed in Table 1.

**Table 1:** List of primers used in real-time PCR

Gene name	Primer	Sequence (5′–3′)
*GAPDH*	Forward	ACTGCCACCCAGAAGACTGT
	Reverse	CCATGCCAGTGAGCTTCC
*BNIP3l*	Forward	CACACCAGCAGGGACCATAG
	Reverse	TCTGCGGAGAAAATACCCCC
*ULK1*	Forward	TTCCAAACACCTCGGTCCTC
	Reverse	CCAACTTGAGGAGATGGCGT
*BECLIN1*	Forward	GAGGTTGAGAAAGGCGAGACA
	Reverse	ATTGTGAGGACACCCAAGCAA
*LC3a*	Forward	CTTCTGAGCCAGCAGTAGGG
	Reverse	CCAGAGGGACAACCCTAACA
*LC3b*	Forward	ACCACACCCAAAGTCCTCAC
	Reverse	CACTGCTGCTTTCCGTAACA
*GABARAPL1*	Forward	AGGAGGACCATCCCTTTGAG
	Reverse	AGTAAGGTCAGAGGGCACTAGG
*GABARAPL2*	Forward	GCGAAGATTCGAGCGAAATA
	Reverse	TGTGGGACTGTCTTATCCACA

### Apoptosis assay

To determine apoptosis, the cells were harvested and stained with AnnexinV/PI. Briefly, the cells were washed and re-suspended in 1× binding buffer (10 mM HEPES, 140 mM NaCl, and 2.5 mM CaCl_2_). Further, they were stained with AnnexinV-FITC (BD Biosciences, San Jose, CA, USA) for 20 min at RT. The cells were again washed to remove excess antibody. Propidium iodide (PI) (Sigma-Aldrich) (50 μg/ml) was added to each sample just before acquiring cells on FACS Canto II (BD, San Jose, CA, USA).

### Statistical analysis

The statistical difference between groups was analyzed either by paired *t*-test or by one-way RM ANOVA using the SigmaPlot software (Version 11.0). Data are presented as mean ± SEM. Results were considered statistically significant for probability values *p ≤ 0.05, **p ≤ 0.01, and ***p ≤ 0.001.

## Results

### Development of a protocol for efficient generation of red blood cells from HSCs

First, we developed and optimized a three-step protocol for the expansion and generation of RBCs from HSCs, as described in the “Materials and methods” section. This is illustrated as a flow chart in Additional file 1: Figure S1a. Using this protocol, RBCs could successfully be generated from HSCs isolated from apheresis-derived peripheral blood (APBL) samples as described below.

#### Low concentration of TGF-β1 does not affect cell proliferation

To determine the optimal concentration of TGF-β1, we incubated the TF1 cell line with different concentrations of TGF-β1 for 24 and 48 h. Cell viability was assessed by MTT assay. Cell viability decreased in a concentration-dependent manner after 24 h and 48 h of TGF-β1 treatment (Additional file 1: Figure S1b). For subsequent experiments, we chose to work with a low concentration of TGF-β1, i.e., 10 pg/ml which was minimally affecting cell proliferation at both the time points.

**Fig. 1:**
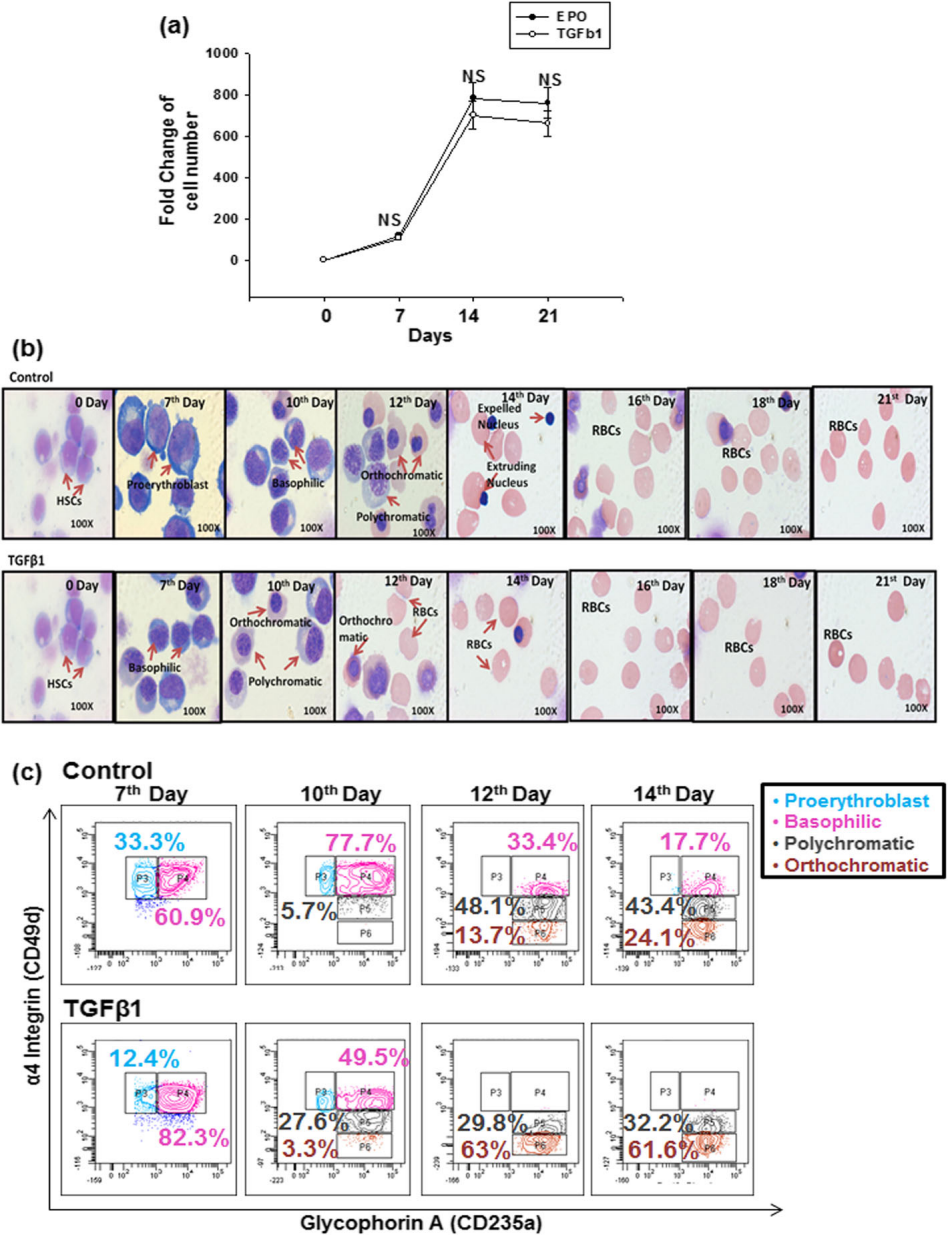
Low TGF-β1 concentration does not affect cell proliferation and induces acceleration of erythroid maturation stages. HSCs derived from APBL were differentiated to erythroid lineage for 21 days with or without addition of TGF-β1. **a** Fold increase in total cell number is depicted. Mean values ± SEM from independent experiments done with cells from three different donors are shown. NS = non-significant. **b** Representative images of Wright’s and Giemsa-stained control and TGF-β1 cultures at different time points, showing the early maturation of erythroid stages by TGF-β1 supplementation. Images were taken at × 100. **c** Representative flow panel showing early erythroid stages of maturation in the presence of TGF-β1

Further, we studied the effect of TGF-β1 at 10 pg/ml concentration on the proliferation of HSCs which were directed towards RBC differentiation. These cells were harvested at different intervals and their viability was assessed by the trypan blue dye exclusion method. As depicted in Fig. 1a, we observed that low concentration of TGF-β1, i.e., 10 pg/ml does not adversely affect the proliferation of early-stage erythropoiesis (7th day), whereas there was a marginal decrease in the late stages of erythropoiesis (days 14 and 21), as compared to the control set, but the difference was not significant.

Taken together, these data suggest that the addition of a low concentration of TGF-β1 does not adversely affect cell proliferation of early-stage erythropoiesis.

#### Low concentration of TGF-β1 accelerated erythroid maturation

TGF-β1 at a concentration of 2 ng/ml and 5 ng/ml is reported to inhibit proliferation and induce acceleration of the late stages of the erythropoiesis process [11, 27]. To examine whether a low concentration of TGF-β1, i.e., 10 pg/ml also has similar effects, we performed a morphological analysis of the cultured cells at various time points. As is evident in Fig. 1b, on day 7, TGF-β1 induces a reduction of proerythroblasts but increases basophilic erythroblasts. On day 10, the control set contained the maximum number of proerythroblasts and basophilic erythroblasts, whereas, in the test set, polychromatic and orthochromatic erythroblasts already started appearing. Similarly, accelerated erythroid differentiation was observed on day 12, day 14, and day 16 in the test set, as compared to the control set. At each time point, around 500 cells were counted and percentages of different erythroid stages were calculated (Additional file 2: Figure S2a). The data showed an acceleration of erythroid stages even after the addition of TGF-β1 at low concentration. This observation was based on manual counting of cells in random fields.

To further validate these data, we performed a phenotypic analysis of the cells. Distinct stages of erythroblasts were differentiated based on the progressive loss of α4 integrin coupled with a progressive increase in the expression of glycophorin A (CD235a) (Additional file 2:Figure S2b). Consistent with the data obtained in morphological characterization, a significantly accelerated maturation of erythroid cells was seen at an earlier time point in the test set, as compared to the control set (Fig. 1c). Data for five different samples showing different stages of erythroid differentiation on days 7, 10, 12, and 14 are represented in Additional file 2:Figure S2c-f.

Overall, these data suggest that the addition of TGF-β1 at 10 pg/ml accelerates the erythroid maturation process.

#### TGF-β1 promotes early maturation leading to an enhanced RBCs production

**Fig. 2:**
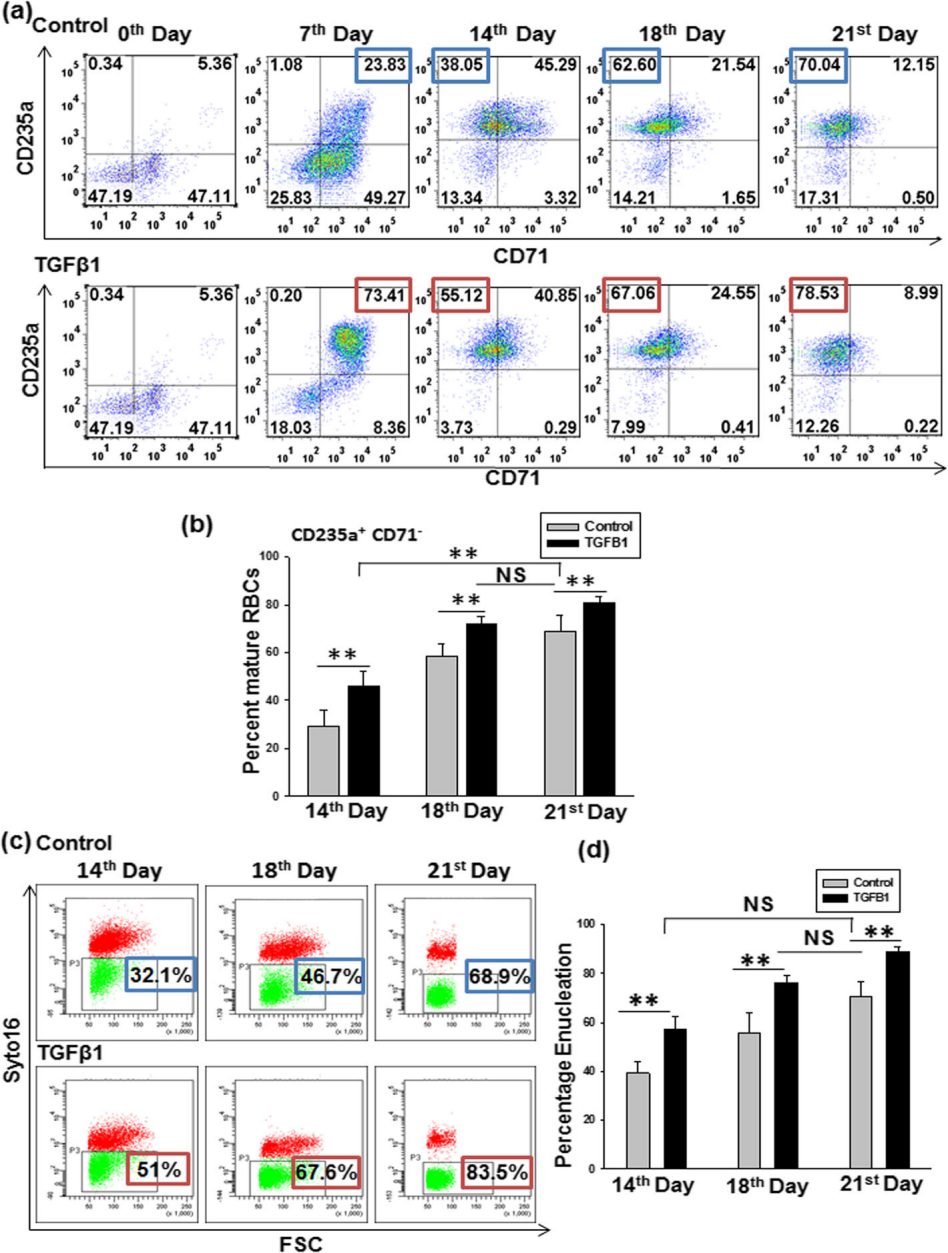
TGF-β1 enhances maturation and enucleation of RBCs. **a** Representative flow panels showing expression of glycophorin A(CD235a) and transferrin receptor (CD71) during erythropoiesis in cultures set with and without TGF-β1. **b** Graph showing percentage of mature RBCs (CD235a^+^CD71^−^) in cell cultures with TGF-β1, as compared to control. Data show mean ± SEM from independent experiments done with cells from seven different donors. **p < 0.01, NS = non-significant. **c** Flow cytometric analysis showing enucleated RBCs. Enucleated RBCs percent is calculated as the percent of SYTO16^−^ cells in the total cells excluding debris and nuclei. **d** Graph showing the percentage of enucleated RBCs in TGF-β1 set, compared to the control set at different time points. Results are presented as mean ± SEM from independent experiments done with five different donor samples. **p < 0.01, NS = non-significant

Commitment towards erythroid lineage is marked by progressive loss of transferrin receptor (CD71) and the slow appearance of glycophorin A (CD235a). This was analyzed by flow cytometry. On day 7, there was a significant increase in immature erythroblast (CD71^+^CD235a^+^) in the test set, as compared to the control set (Fig. 2a). On days 14, 18, and 21 TGF-β1 supplementation caused a significant enhancement in the percentage of mature RBCs (CD71^−^CD235a^+^), as compared to the control set. Data for seven different samples showed that TGF-β1 addition causes a significant enhancement in RBC formation as confirmed by an increased expression of CD235a and a reduced expression of CD71 at a much earlier time point, as compared to the control set (Fig. 2b). Moreover, we observed that on the 14th day, the yield of percent mature RBCs in the TGF-β1 set was less than that in the control set on the 21st day. But on the 18th day, set this yield in the TGF-β1 (72 ± 2.79%) was equivalent to the 21st day in the control set (68.77 ± 6.58%).

These data indicate that TGF-β1 accelerates and enhances the maturation of RBCs.

#### Low concentration of TGF-β1 increases enucleation of RBCs

Enucleation is the last step in the terminal differentiation of RBCs. Percentage enucleation was assessed by nucleic acid binding dye SYTO16 along with forward scatter (FSC) representing cell size. By combining SYTO16 with FSC, SYTO16^+^FSC^high^, SYTO16^−^FSC^high^, and SYTO16^+^FSC^low^ were identified as nucleated erythroblasts, enucleated RBC, and extruded nuclei, respectively (Additional file 3:Figure S3a). From day 14, the addition of TGF-β1 resulted in a significant increase in the percent of enucleated RBCs (57.48 ± 4.72 in the test set versus 39.18 ± 4.94 in the control set) (Fig. 2c). This rise in the number of enucleated RBC continued till day 21 in the test set (88.82 ± 1.93), as compared to the control set (70.44 ± 6.2). In five independent experiments, TGF-β1 addition caused a significant increase in the percent of enucleated RBCs as monitored on days 14, 18, and 21, supporting the role of TGF-β1 in enhancing terminal enucleation process (Fig. 2d).

Additionally, we observed that percent enucleated RBCs in the TGF-β1 set on day 14 (57.48 ± 4.72) and day 18 (76.1 ± 3.22%) was comparable to that generated on the 21st day in the control set (70.44 ± 6.2%).

Since the percentage of both mature and enucleated RBCs on day 18 in the TGF-β1 set was equivalent to day 21 control set RBCs, our data suggest that the incorporation of TGF-β1 at a concentration of 10 pg/ml accelerates and enhances erythropoiesis process by 3 days.

### RBCs generated after TGF-β1 supplementation are equivalent to normal adult RBCs

Here we examined whether the RBCs generated in the presence of TGF-β1 resembled normal RBCs by subjecting them to various assays as described below.

#### TGF-β1 does not affect the synthesis of hemoglobin chain

**Fig. 3:**
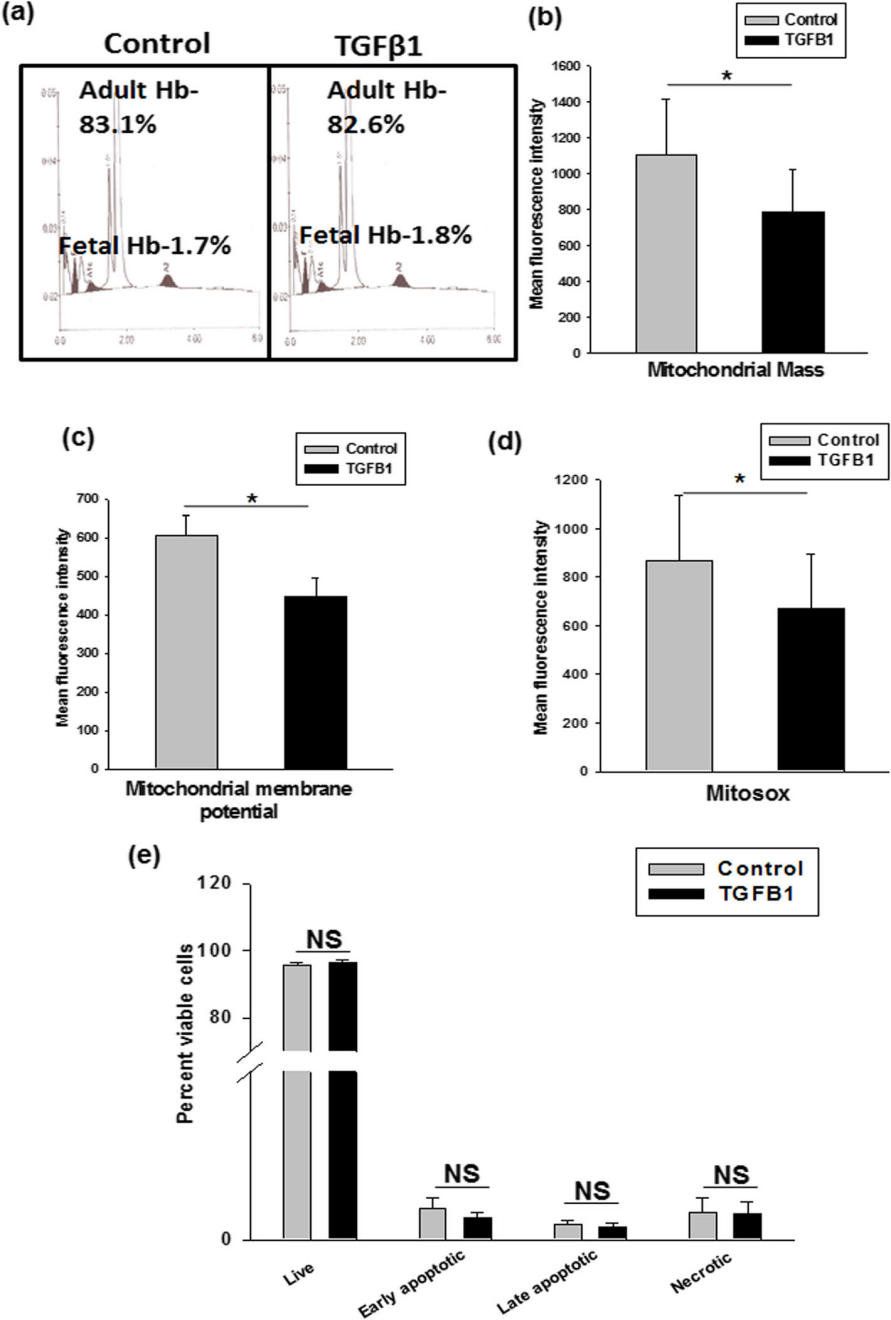
HPLC analysis of generated RBCs and TGF-β1 promotes mitochondrial clearance during terminal erythroid differentiation. **a** HPLC analysis showing the hemoglobin content in the in vitro generated RBCs. TGF-β1 supplementation significantly decreases **b** mitochondrial mass, **c** mitochondrial membrane potential, **d** mitochondrial ROS, as compared to control set on day 12. Results are presented as mean ± SEM from independent experiments with five different donor samples.*p ≤ 0 .05. **e** The graph shows that TGF-β1 does not induce apoptosis in cultured cells. Mean values ± SEM from three different donor samples are shown. NS = non-significant

The hemoglobin content of generated RBC was analyzed by HPLC. We found that at day 21 the adult, as well as fetal hemoglobin in the TGF-β1 set, was comparable to that seen in the control set (Fig. 3a). Cumulative data for three different independent experiments show that TGF-β1 does not adversely affect the synthesis of hemoglobin chains (Table 2).

**Table 2:** Adult and fetal hemoglobin content of RBCs from control and TGF-β1 sets

21st day	Control	TGFβ1
Adult hemoglobin	79.36 ± 4.45	80.33 ± 1.32
Fetal hemoglobin	2.26 ± 0.42	1.26 ± 0.63

Legend: Table showing adult and fetal hemoglobin content of RBCs from control and TGF-β1 sets. Data show mean ± SEM from independent experiments done with cells from three different donors

#### TGF-β1 does not affect hematocrit parameters

Various hematocrit parameters of RBCs such as mean corpuscular volume (MCV), mean cell hemoglobin (MCH), and mean corpuscular hemoglobin concentration (MCHC) were analyzed using an automated coulter counter. On day 21, cells in test set displayed MCV, MCH, and MCHC values equivalent to that of the control set (Table 3). All these parameters of control, as well as the test set, were only marginally higher than those of normal adult peripheral blood RBCs.

**Table 3:** Hematocrit parameters of cultured RBCs

Hematocrit parameter	21st day		Normal range
	Control	TGFβ1	
Mean corpuscular value (fL)	131.00 ± 5.85	133.33 ± 8.33	90–100
Mean cell hemoglobin (pg)	37.66 ± 2.33	45.66 ± 4.33	27–33
Mean corpuscular hemoglobin concentration (g/dL)	28.6 ± 2.33	34.33 ± 2.96	33–36

Legend: Table showing hematocrit parameters of RBCs from control and TGF-β1 sets. Data are represented as mean ± SEM from independent experiments with three different donor samples

#### Glucose-6-phosphate dehydrogenase content of RBCs

In RBCs, the presence of glucose-6-phosphate dehydrogenase (G-6-PD) is required for the regeneration of reduced glutathione. A decrease in the level of glutathione results in an increased oxidative stress causing hemolysis of RBCs, thereby leading to anemic condition [28]. We observed that glucose-6-phosphate dehydrogenase (G6PD) content of control and test sets was 35 ± 3.73 and 38 ± 6.50 units per gram of hemoglobin, respectively (Table 4).

**Table 4:** Glucose-6-phosphate dehydrogenase (G6PD) levels of generated RBCs

21st day	G6PD (units/g Hb)
Control	35 ± 3.73
TGFβ1	38 ± 6.50

Legend: Table showing glucose-6-phosphate dehydrogenase (G6PD) levels in cultured RBCs of control and test set. Data are shown as mean ± SEM from independent experiments with three different donor samples

Collectively, these data show that addition of low concentrations of TGF-β1 generates normal mature RBCs.

### Low TGFβ1 accelerates erythropoiesis by arresting the cells at G0/G1 phase

To understand the mechanism by which TGF-β1 enhances the erythropoiesis, we monitored the cell cycle profile of cultured cells. TGF-β1 at a concentration of 2 ng/mL is reported to induce cell cycle arrest of adult CD36^+^ cells which were differentiated towards erythroid lineage [11]. Therefore, we examined whether a low concentration of TGF-β1 was also inducing cell cycle arrest, which is tightly associated with terminal erythroid differentiation. Since enucleation was observed at day 14 onwards (Fig. 2c), we selected days 10 and 12 for cell cycle analysis of erythroblasts. We found that low concentration of TGF-β1 significantly increases the G0/G1 phase and decreases S phase, whereas there was no effect on the G2M phase (Additional file 4:Figure S4a,b and c).

Cyclin-dependent kinase inhibitors (CDKIs) inhibit the activity of cyclin-dependent kinases (CDKs) and promote the cell cycle arrest. Hence, we checked the expression of CDKI p27 and p21 in the TGFβ1-treated cells. We found that there was a significant increase in the expression of p27 in the TGF-β1 set, as compared to the control set (Additional file 4:Figure S4d and e), whereas there was low expression of p21 (Additional file 4:Figure S4f and g).

These data suggest that TGF-β1 arrests the cells at G0/G1 phase by upregulating the expression of p27 CDKI, leading to an enhancement in the percent enucleation of RBCs.

### TGF-β1 increases mitophagy during erythropoiesis process

Autophagy is particularly involved in the clearance of mitochondria (mitophagy) during terminal erythroid maturation [21–24]; therefore, we investigated whether an increased mitophagy was involved in the TGF-β1-mediated enhancement in erythroid differentiation. Autophagy is induced at polychromatic stages [18]. Hence, we selected day 12 for subsequent experiments, wherein late stages of erythropoiesis, i.e., polychromatic and orthochromatic erythroblasts were present in both, control and test sets. To assess the mitochondrial content during erythroid differentiation, we first quantified mitochondrial mass by using mitotracker green. Notably, we observed a significant decrease in mean fluorescence intensity (MFI) of mitochondrial mass in the TGF-β1 set (785 ± 236.13) as compared to the control set (1104 ± 313.91) (Fig. 3b).

A loss of mitochondrial membrane potential triggers the onset of mitophagy. TMRE dye was used to track mitochondrial membrane depolarization. The data showed that there was a significant decrease in MFI of mitochondrial membrane potential in the test set (445 ± 48.77), as compared to the control set (605 ± 51) demonstrating a decrease in mitochondrial activity (Fig. 3c). This was further supported by a decrease in mitochondrial ROS production in the TGF-β1 set, as detected by MitoSOX staining (Fig. 3d). In the case of impaired mitophagy, an accumulation of damaged mitochondria and an increase in ROS levels may lead to apoptosis and ineffective erythropoiesis [29]. We observed that there was no change in the apoptosis level in the cells from both control and test sets (Fig. 3e). Representative flow cytometry plots for mitochondrial mass, membrane potential, MitoSOX, and apoptosis are shown in Additional file 5:Figure S5a-d.

These data suggest that the addition of TGF-β1 facilitates the clearance of mitochondria during terminal erythroid differentiation.

#### TGF-β1 augments expression of mitophagy genes during terminal erythroid differentiation

**Fig. 4:**
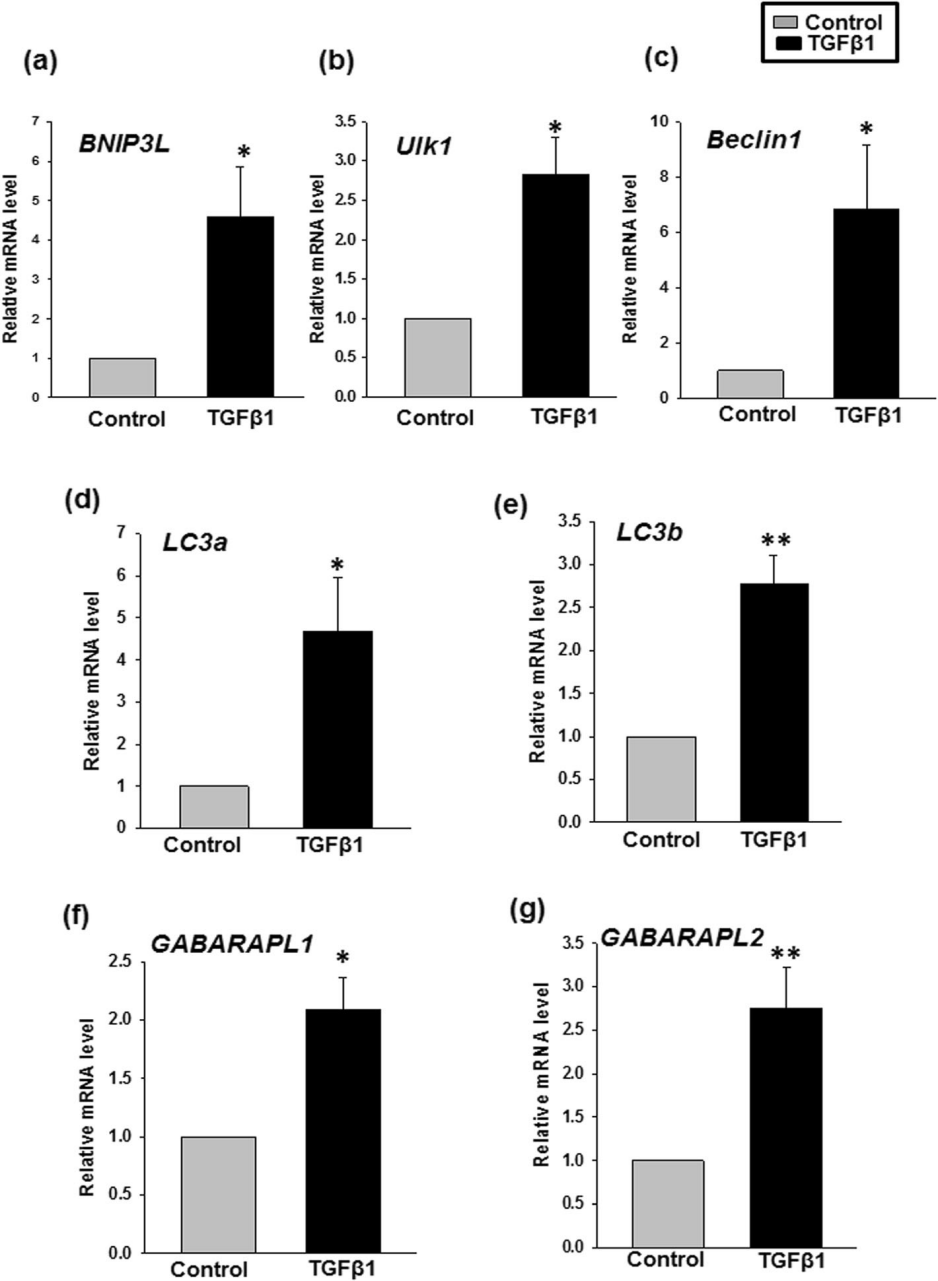
TGF-β1 regulates mitophagy gene expression during terminal erythroid differentiation. Cultured cells from day 12 of control and TGF-β1 set were subjected to quantitative reverse transcriptase-polymerase chain reaction (qRT-PCR) analysis to quantify the mitophagy-inducing mRNAs. Cells cultured with TGF-β1 show a significant increase in the level of **a***BNIP3L*, **b***Ulk1*, **c***Beclin1*, **d***LC3a*, **e***LC3b*, **f***GABARAPL1*, and **g***GABARAPL2*. Results are presented as mean ± SEM from independent experiments done with five different donor samples. *p < 0.05; **p < 0.01

We further validated our data by determining the expression of mitophagy-related genes in the cultured cells by real-time PCR. BNIP3L/NIX is a known outer mitochondrial membrane protein involved in the induction of mitophagy during terminal erythroid differentiation [22, 30]. The expression of BNIP3L/NIX in the test set was found to be 3.58-fold greater than that of the control set (Fig. 4a). We also found a significant upregulation of Ulk1 and Beclin1 in the test set (Fig. 4b, c). This finding is supported by the known function of these genes in activating the autophagy pathway needed for mitochondrial removal [31–33]. Similarly, we also observed a significant upregulation of other autophagy-related genes such as LC3a/LC3b and GABARAPL1/GABARAPL2 in the TGF-β1 supplemented set (Fig. 4d–g). Therefore, these findings support the role of TGF-β1 in promoting mitophagy possibly by BNIP3L/NIX pathway during erythroid differentiation.

#### TGF-β1-mediated increase in mitophagy leads to enhanced erythroid differentiation

**Fig. 5:**
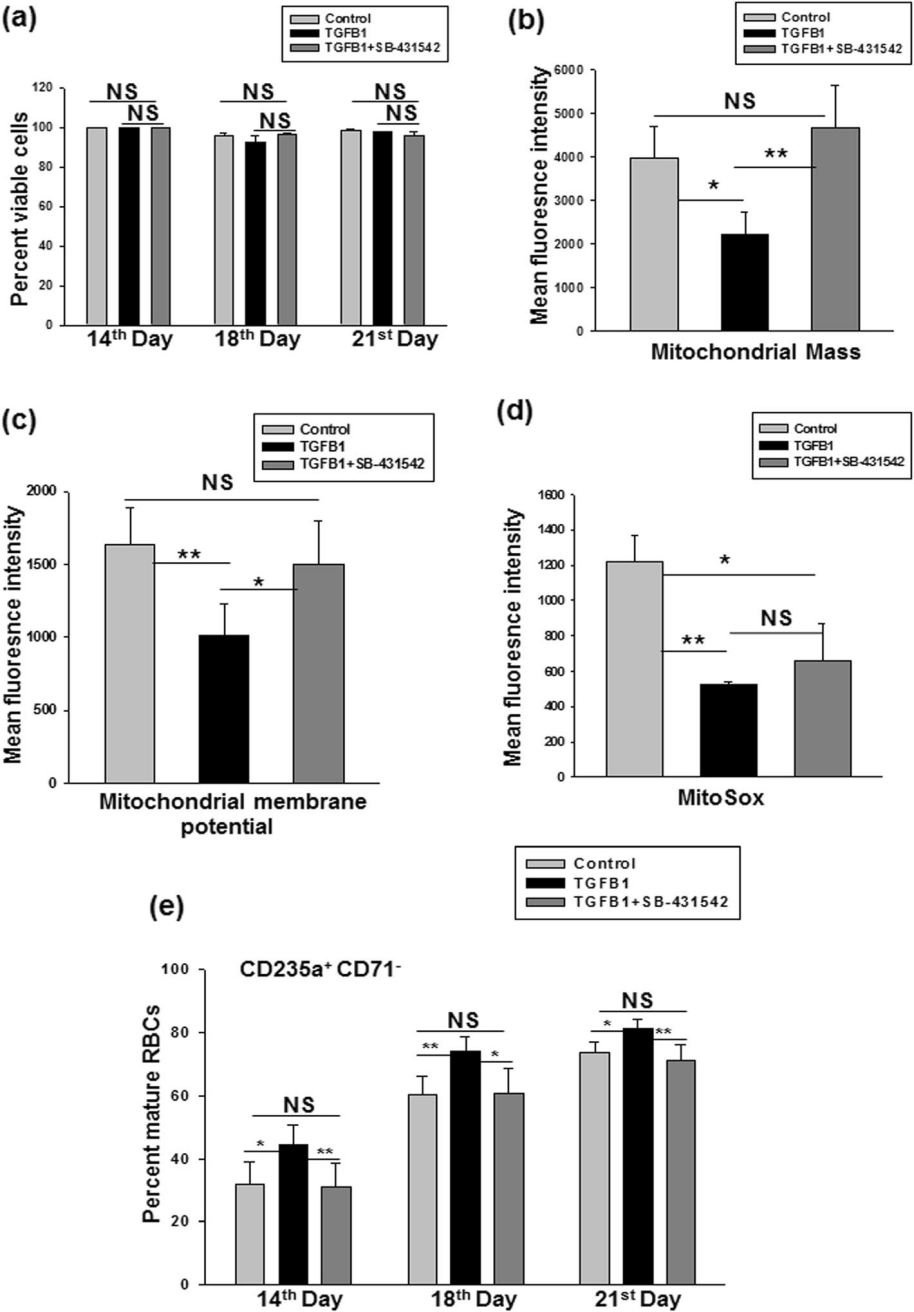
TGF-β1 enhances terminal erythroid differentiation through the regulation of mitophagy pathway. **a** TGF-β1 inhibitor (SB-431542) does not affect the viability of cells on days 14, 18 and 21 as determined by Calcein Am staining. Results are presented as mean ± SEM from data obtained in independent experiments done using three different donor samples. NS = non-significant. SB-431542 treatment in TGF-β1 set increases **b** mitochondrial mass, **c** mitochondrial membrane potential to control level, and **d** mitochondrial ROS level on day 12 after SB-431542 treatment. Data show mean ± SEM from independent experiments done with cells from five different donors. *p < 0.05; **p < 0.01; NS = non-significant. **e** Percent of mature RBCs returned to the percentage of control set after SB-431542 treatment on days 14, 18, and 21. Results are represented as mean ± SEM from independent experiments performed using five different donor samples. *p < 0.05; **p < 0.01; NS = non-significant

To determine whether the TGF-β1-mediated increase in mitophagy causes an enhancement in the terminal erythroid differentiation, we evaluated the effect of an inhibitor of TGF-β1 receptor, SB-431542. TGF-β1 inhibitor at a concentration of 1 μM was added in TGF-β1 set during the late stages of erythropoiesis, i.e., from day 7 onwards. As seen in Fig. 5a, the addition of SB-431542 did not affect the viability of the cells throughout the culture period as assessed by Calcein Am staining. We observed that on day 12, mitochondrial mass and mitochondrial membrane potential significantly increased in the SB-431542 treatment set, as compared to the TGF-β1 set, and almost reached the same level as the control set (Fig. 5b,c). Likewise, mitochondrial ROS level after SB-431542 treatment showed an increase in trend, similar to the control set, but the difference failed to reach statistical significance (Fig. 5d).

We further show that SB-431542 treatment in TGF-β1 set significantly decreases RBC production and brings it back to the level of control set on days 14, 18, and 21 (Fig. 5e). Representative flow cytometry overlays for cell viability, mitochondrial mass, membrane potential, MitoSOX, and percent mature RBCs are shown in Additional file 6: Figure S6a-e.

These data confirm that the exogenous addition of TGF-β1 promotes erythropoiesis by promoting mitophagy.

Collectively, our data demonstrate that TGF-β1 enhances terminal erythroid differentiation through the regulation of mitophagy and p27-mediated cell cycle arrest.

## Discussion

RBC transfusion is a life-saving treatment in numerous therapies. A widespread shortage of blood supply, mostly in developing countries, has led to the development of alternative blood products. Many attempts are being made worldwide for ex vivo generation of blood cells from different stem cell sources [34]. As far as the in vitro generation of RBCs is concerned, several hurdles remain in obtaining clinical-grade culture RBCs. Therefore, major research efforts are directed towards the refinement of protocols for their generation. However, lengthy culture time needed to generate RBCs in vitro adds the extra cost of culture medium, growth factors and other reagents, which may impede the clinical application of in vitro generated RBCs [35–37]. Acceleration of the in vitro erythropoiesis process by using small molecules may reduce the cost and eventually make the large-scale production of these cells affordable.

One such molecule is the cytokine-TGF-β1, which is reported to accelerate terminal erythroid differentiation. An earlier study has shown the effect of TGF-β1 at a concentration of 2 ng/ml on the UT7 cell line, wherein it was shown to accelerate erythroid differentiation as assessed by induction of glycophorin expression and hemoglobin synthesis [10]. Another study has reported that TGF-β1 (2 ng/ml) is a paraxodial inhibitor of erythropoiesis which acts by inhibiting proliferation and acceleration of differentiation of CD36^+^ erythroid progenitors [11]. Similarly, Salem Akel et al. reported that TGF-β1 at 5 ng/ml concentration accelerates terminal erythroid differentiation of erythroid progenitors with enucleation percent of 54% ± 18% [27]. Our study is quite distinct from these previous reports on several accounts. Firstly, in these studies, nanogram concentrations of TGF-β1 have been used; secondly, the effect is either studied on a cell line or on erythroid progenitors; thirdly, our protocol is distinct from the reported ones; and, lastly and importantly, they have not reported enhancement in the formation of mature enucleated RBCs.

The effect of TGF-β1 on cell proliferation and differentiation depends on its concentration and the cell type used. Earlier, it was reported that TGF-β1 at low concentration (10–20 pg/ml) significantly stimulated colony formation from HSCs, indicating that these progenitors are the direct target of stimulatory action of TGF-β1, whereas at high TGF-β1 concentration (5–10 ng/ml) it inhibited colony formation [16, 17, 38]. Here, we show, for the first time, that low concentration of TGF-β1 is more effective on erythroid differentiation of HSCs. TGF-β1 at picogram concentration showed no inhibition of proliferation on the early stages of erythropoiesis till day 7. On days 14 and 21, though there was a decrease in proliferation of late-stage erythropoiesis in the TGF-β1 set, the difference was marginal. This decrease in the rate of proliferation might be due to the arrest of the cell cycle of immature erythroid cells in favor of differentiation which is in agreement with an earlier report [11].

Early erythropoiesis involves the proliferation of HSCs and their differentiation into erythroid burst forming a unit (BFU-E) and erythroid colony-forming unit (CFU-E). Terminal erythroid differentiation begins with first recognizable erythroid progenitors, i.e., proerythroblasts. These proerythroblasts further undergo sequential mitoses to form basophilic, polychromatic, orthochromatic erythroblasts, and finally, mature RBCs. We observed that the addition of TGF-β1 from the initial day of the culture system helps in preponing the entire erythropoiesis process as assessed by morphological and phenotypical analysis. These data indicate that TGF-β1 not only acts on late erythroid stages but also acts on the early stages of erythroid maturation. Gao et al. demonstrated that human cord blood CD34^+^-derived BFU-E and CFU-E express low to a high level of transforming growth factor III receptor (TGFβIII receptor) respectively [39]. TGFβIII receptor has an affinity to bind to all isoforms of TGF-β1. In our culture system, the exogenous addition of TGF-β1 might be helping in binding to this receptor at even the earlier stages, resulting in the initiation of the TGF-β1-mediated signaling pathway. This might be one of the possible mechanisms of how TGF-β1 is accelerating the erythropoiesis process. However, one needs to confirm this by further investigations. This study is being pursued in our lab.

Previous report highlighting the TGF-β1-mediated increase in erythroid differentiation of terminal erythroid progenitors was based only on the induction of glycophorin A (CD235a) expression and not on the expression of transferrin receptor [11]. However, mature RBCs are defined as being CD235a^+^CD71^−^ and percent enhancement of this population by TGF-β1 was not examined. Here we found that low concentration of TGF-β1 significantly increased the number of immature erythroblasts (CD235a^+^CD71^+^) on day 7, and a significant enhancement in the production of mature RBCs (CD235a^+^CD71^−^) was seen from day 14 onwards. Similarly, we also got a significant enhancement in the percent enucleation. Yael Zermati et al. and Salem Akel et al. reported 45 ± 4% and 54 ± 18% of enucleated RBCs when erythroid progenitors were treated with 2 ng/ml and 5 ng/ml of TGF-β1, respectively [11, 27]. In the present study, we succeeded in obtaining more than 85% of enucleated cells by day 21.

One point of concern could be that the acceleration of the in vitro erythropoiesis process may lead to the generation of defective RBCs. To address this issue, we determined hemoglobin content, hematocrit parameter, and glucose-6-phosphate dehydrogenase content. Hemoglobin content was almost identical to the control RBCs. Hematocrit parameter and G-6-P dehydrogenase content were also identical to the control set RBCs, although the values were slightly higher than the adult normal RBCs. This variation might be due to in vitro culture conditions and need further optimization. However, it is now very well established that ex vivo generated reticulocytes mature further when infused in vivo, resulting in a reduction in surface area and subsequently acquiring a typical biconcave shape [3]. Hence, these parameters could get normalized after in vivo infusion. However, this needs to be formally examined.

One of the known mechanisms of accelerating erythroid differentiation by TGF-β1 involves a reduction in the proliferation of intermediate and late erythroid progenitors and their consequent accelerated differentiation by skipping cell division [11]. Similarly, we also found that the low concentration of TGF-β1 arrests the cell cycle at the G0/G1 phase and also significantly reduces the S phase. TGF-β1 is reported to induce G1 cell cycle arrest by upregulating the expression of CDKI p15, p21, and p27 in different cell types [40–42]. We observed that the supplementation of low TGF-β1 concentration significantly increases p27 level. An earlier report showed an undetectable level of p15 and a low level of p21 during terminal stages of erythropoiesis at the RNA level [43]. In agreement with their data, we also observed less expression of p21 at the protein level. These data indicate that TGF-β1 accelerates enucleation process by p27-mediated cell cycle arrest.

Additionally, as we obtained a significant enhancement in the percentage of mature RBCs and their enucleation from day 14 in the TGF-β1 set, we also focused on the mechanism, which specifically acts at the final stages of erythropoiesis. Previous studies have shown the importance of mitophagy, a specific autophagic process involved in mitochondrial clearance at the final maturation stage of RBCs [21–24]. However, the role of TGF-β1 in inducing mitophagy during terminal erythroid differentiation has not been explored. Mitochondrial membrane depolarization is a potent stimulus for the initiation of mitophagy. BNIP3L/NIX is the outer mitochondrial membrane protein that gets upregulated during terminal erythroid differentiation. This protein causes mitochondrial membrane depolarization leading to recognition and sequestration of mitochondria by autophagosomes [22, 30]. Here we show, for the first time, that TGF-β1 supplementation reduces mitochondrial mass, mitochondrial membrane potential, and ROS generation indicating that TGF-β1 is promoting mitochondrial clearance. Moreover, we also reported the upregulation of genes involved in mitophagy and autophagy process in TGF-β1 set during terminal differentiation.

Furthermore, we also showed that TGF-β1 inhibitor treatment (SB-431542) in the TGF-β1 supplementation set significantly increased both mitochondrial mass and mitochondrial membrane potential, indicative of reduced mitophagy, and decreased the mature RBC production to that in the control set. Therefore, these data suggest that TGF-β1 might be promoting the expression of genes involved in mitophagy, thereby enhancing the in vitro maturation of RBCs. Kiyono et al. demonstrated that TGF-β1 induces the autophagy pathway through both Smad and non-Smad signal transduction pathways in human hepatocellular carcinoma cell lines [26]. TGF-β1 is known to bind to TGFβI and TGFβII receptor. TGF-β1 binds to TGFβI receptor and triggers the Smad pathway. We observed that the inhibitor of TGFβI receptor (SB-431542) attenuated both the mitophagy and the production of mature RBCs. These data suggest the involvement of the Smad pathway in inducing mitophagy during terminal stages of erythroid differentiation. However, it would be interesting to examine whether TGF-β1 also induces the non-Smad signaling pathway for promoting mitophagy during terminal erythropoiesis process. This aspect is included in our future studies.

## Conclusion

In summary, our data demonstrate that a low concentration of TGF-β1 does not inhibit the proliferation of the early stages of erythropoiesis. Additionally, it significantly accelerates the terminal erythropoiesis process leading to an early generation of normal mature enucleated RBCs by promoting BNIP3L/NIX-mediated mitophagy. Overall, our data suggest that in vitro RBC formation can be hastened up by 3 days using a low concentration of TGF-β1.

This important finding could have potential applications in the field of clinical-grade RBC generation for transfusion purposes.

## Supplementary information

Additional file 1: **Figure S1.** Protocol for in vitro RBCs generation and cell viability assessed by MTT assay. a) Flow chart showing protocol used for in vitro generation of RBCs from HSCs obtained from APBL. b) Cell viability of TF1 cell line was evaluated by MTT assay during 24 h and 48 h of TGF-β1 treatment.

Additional file 2: **Figure S2.** TGF-β1 accelerates erythroid maturation. a) The graph shows the percentages of distinct erythroid stages at different time points determined by counting 500 cells from random fields of smears stained with Wright’s and Giemsa stain. b) Representative flow panel shows CD235a and α4integrin staining profile of cultured cells and corresponding images of cells stained with Wright’s and Giemsa stain. Graph showing different stages of erythroid differentiation characterized by phenotypic analysis on c) day 7 d) day 10 e) day 12 and f) day 14. Results are presented as mean ± SEM from independent experiments with five different donor samples. *p < 0.05; **p < 0.01; ***p < 0.001; NS=Non significant.

Additional file 3: **Figure S3.** Flow cytometry analysis of enucleation by SYTO16 staining. a) A representative flow panel showing SYTO16 profile of cultured cells and corresponding images of cells stained with Wright’s and Giemsa stain.

Additional file 4: **Figure S4.** TGF-β1 causes cell cycle arrest during erythropoiesis process. a) Representative flow cytometry overlay showing cell cycle profile of control and TGF-β1 set on day 10 and day 12. TGF-β1 supplementation significantly increases the G0/G1 phase and decreases the S phase on b) day 10 and c) day 12. Data show mean ± SEM from independent experiments done with cells from four different donors. *p < 0.05; **p < 0.01; NS=Non significant. d) Representative flow panel showing mean fluorescence intensity of p27. e) Graph shows an increase in mean fluorescence intensity of p27 in TGF-β1 set compared to the control set on day 10. Results are presented as mean ± SEM from independent experiments with four different donor samples **p < 0.01. f) Representative flow panel showing mean fluorescence intensity of p21. g) The graph shows TGF-β1 supplementation does not affect mean fluorescence intensity of p21 on day 10. Data show mean ± SEM from independent experiments done with cells from four different donors NS=Non significant.

Additional file 5: **Figure S5.** TGF-β1 induces mitophagy in cultured cells. Representative overlays showing a decrease in a) Mitochondrial mass b) Mitochondrial membrane potential and c) Mitochondrial ROS in TGF-β1 set as compared to the control set. MFI: Mean fluorescence intensity. d) Representative dot plot showing apoptosis level of day 12 cultured cells.

Additional file 6: **Figure S6.** TGF-β1 enhances RBC production by inducing mitophagy. Representative flow cytometry overlay showing a) Cells viability by Calcein Am staining b) Mitochondrial mass c) Mitochondrial membrane potential and d) Mitochondrial ROS after SB-431542 treatment. e) Dot plot showing a significant decrease in percent mature RBCs on days 14, 18 and 21 after SB-431542 treatment in TGF-β1 set.

## Abbreviations

APBL: Apheresis-derived peripheral blood
BNIP3L/NIX: BCL2 and adenovirus E1B 19 kDa-interacting protein 3-like
EPO: Erythropoietin
GABARAPL1/2: GABA Type A Receptor Associated Protein Like 1/2
GAPDH: Glyceraldehyde 3-phosphate dehydrogenase
HSCs: Hematopoietic stem cells
IL-3: Interleukin-3
IMDM: Iscove’s modified Dulbecco’s medium
LC3a/b: Microtubule-associated proteins 1A/1B light chain 3A/B
RBCs: Red blood cells
SCF: Stem cell factor
TGF-β1: Transforming growth factor β1
ULK1: Unc-51 like autophagy activating kinase1
