# Supplementary figures and images for: Transforming growth factor β1 accelerates and enhances in vitro red blood cell formation from hematopoietic stem cells by stimulating mitophagy
Source: Stem Cell Res Ther. 2020 Feb 19;11:71. doi: 10.1186/s13287-020-01603-z (PMC7029523; doi:10.1186/s13287-020-01603-z)

**Figure S1****(a)**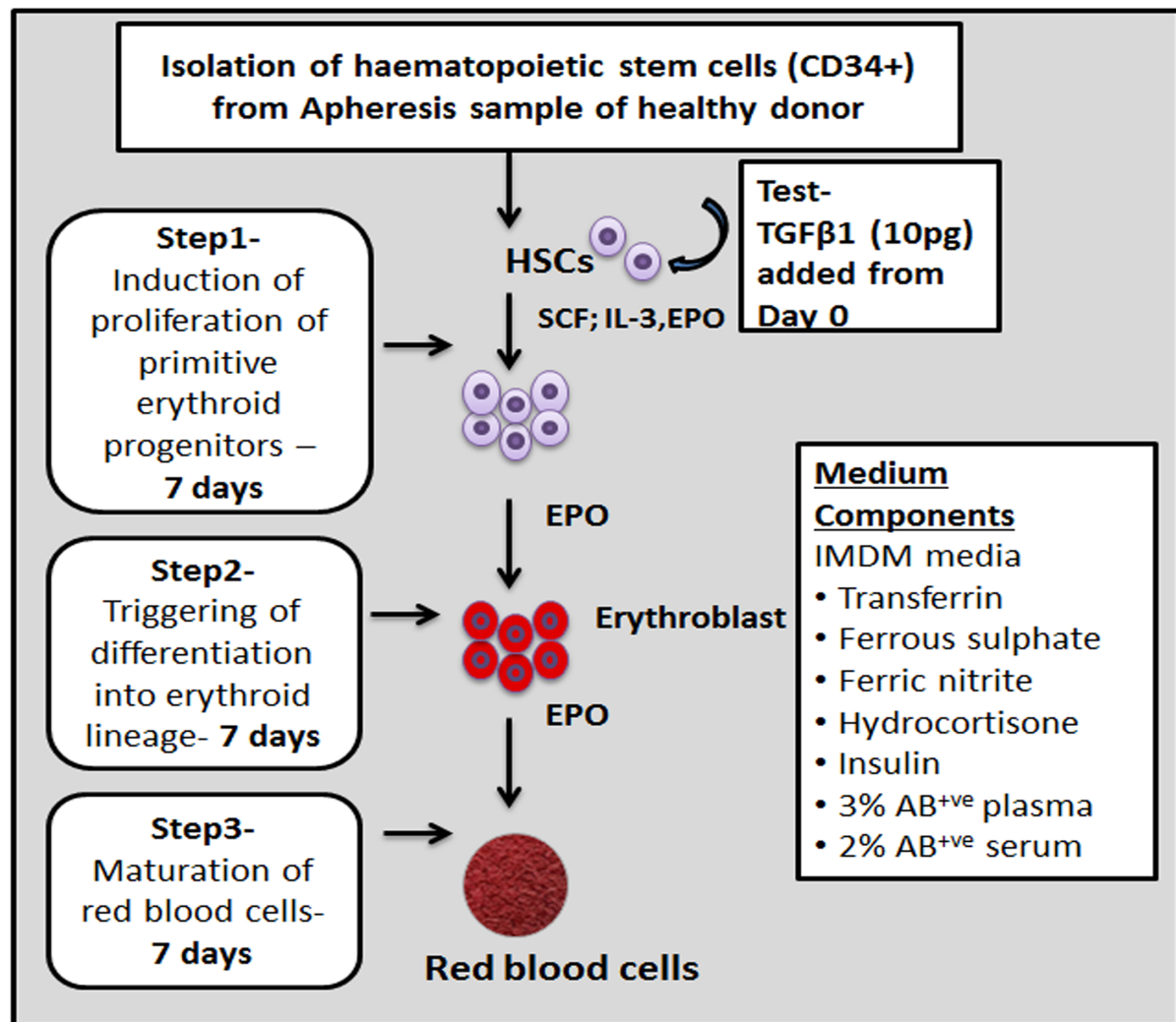**(b)**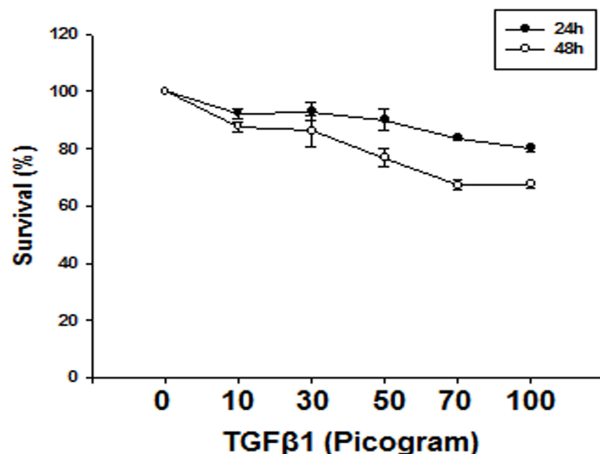

Supplement: Supplementary file 1 — Figure S1. Protocol for in vitro RBCs generation and cell viability assessed by MTT assay. a) Flow chart showing protocol used for in vitro generation of RBCs from HSCs obtained from APBL. b) Cell viability of TF1 cell line was evaluated by MTT assay during 24 h and 48 h of TGF-β1 treatment. [file 13287_2020_1603_MOESM1_ESM.pdf]

**Figure S2**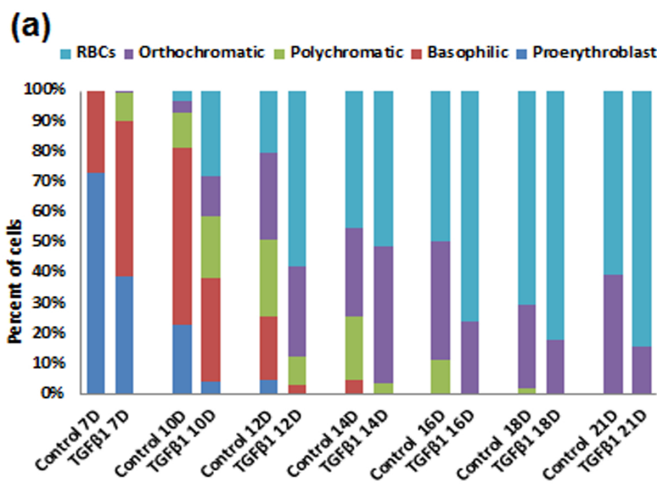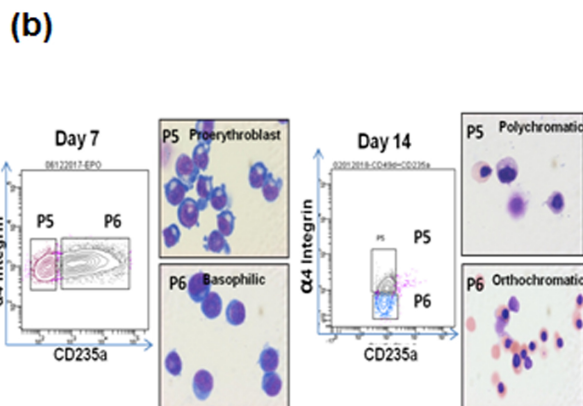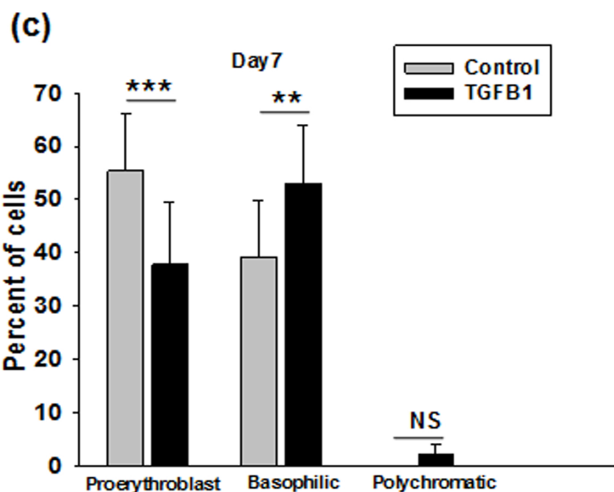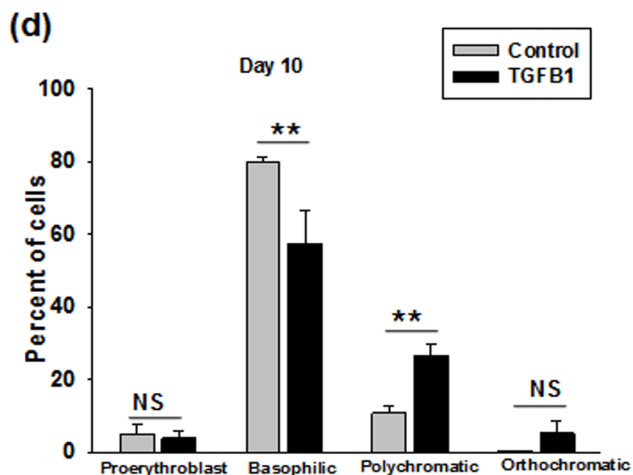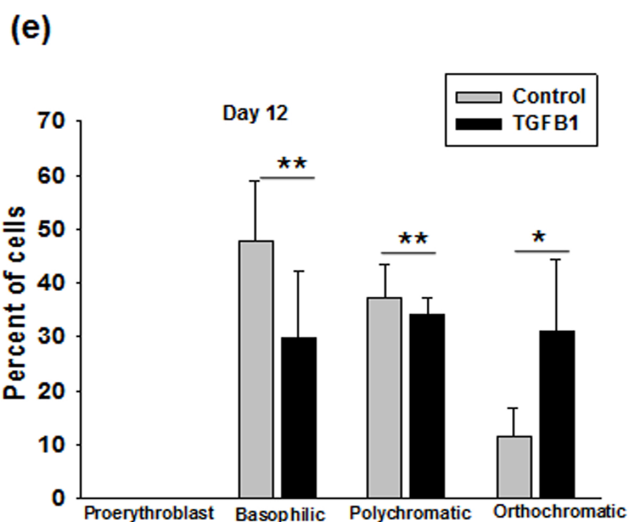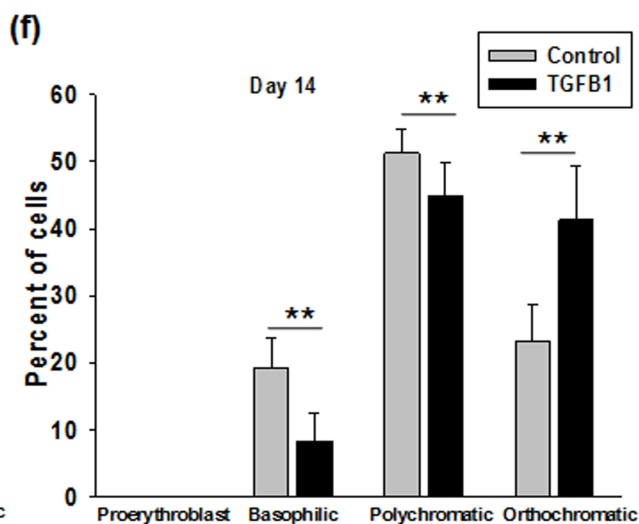

Supplement: Supplementary file 2 — Figure S2. TGF-β1 accelerates erythroid maturation. a) The graph shows the percentages of distinct erythroid stages at different time points determined by counting 500 cells from random fields of smears stained with Wright’s and Giemsa stain. b) Representative flow panel shows CD235a and α4integrin staining profile of cultured cells and corresponding images of cells stained with Wright’s and Giemsa stain. Graph showing different stages of erythroid differentiation characterized by phenotypic analysis on c) day 7 d) day 10 e) day 12 and f) day 14. Results are presented as mean ± SEM from independent experiments with five different donor samples. *p < 0.05; **p < 0.01; ***p < 0.001; NS=Non significant. [file 13287_2020_1603_MOESM2_ESM.pdf]

**Figure S3**

**(a)**

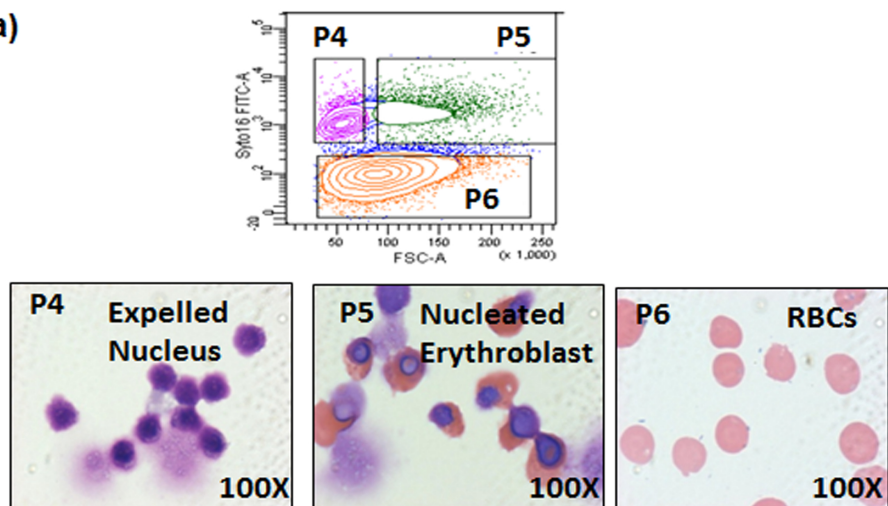

Supplement: Supplementary file 3 — Figure S3. Flow cytometry analysis of enucleation by SYTO16 staining. a) A representative flow panel showing SYTO16 profile of cultured cells and corresponding images of cells stained with Wright’s and Giemsa stain. [file 13287_2020_1603_MOESM3_ESM.pdf]

**Figure S4**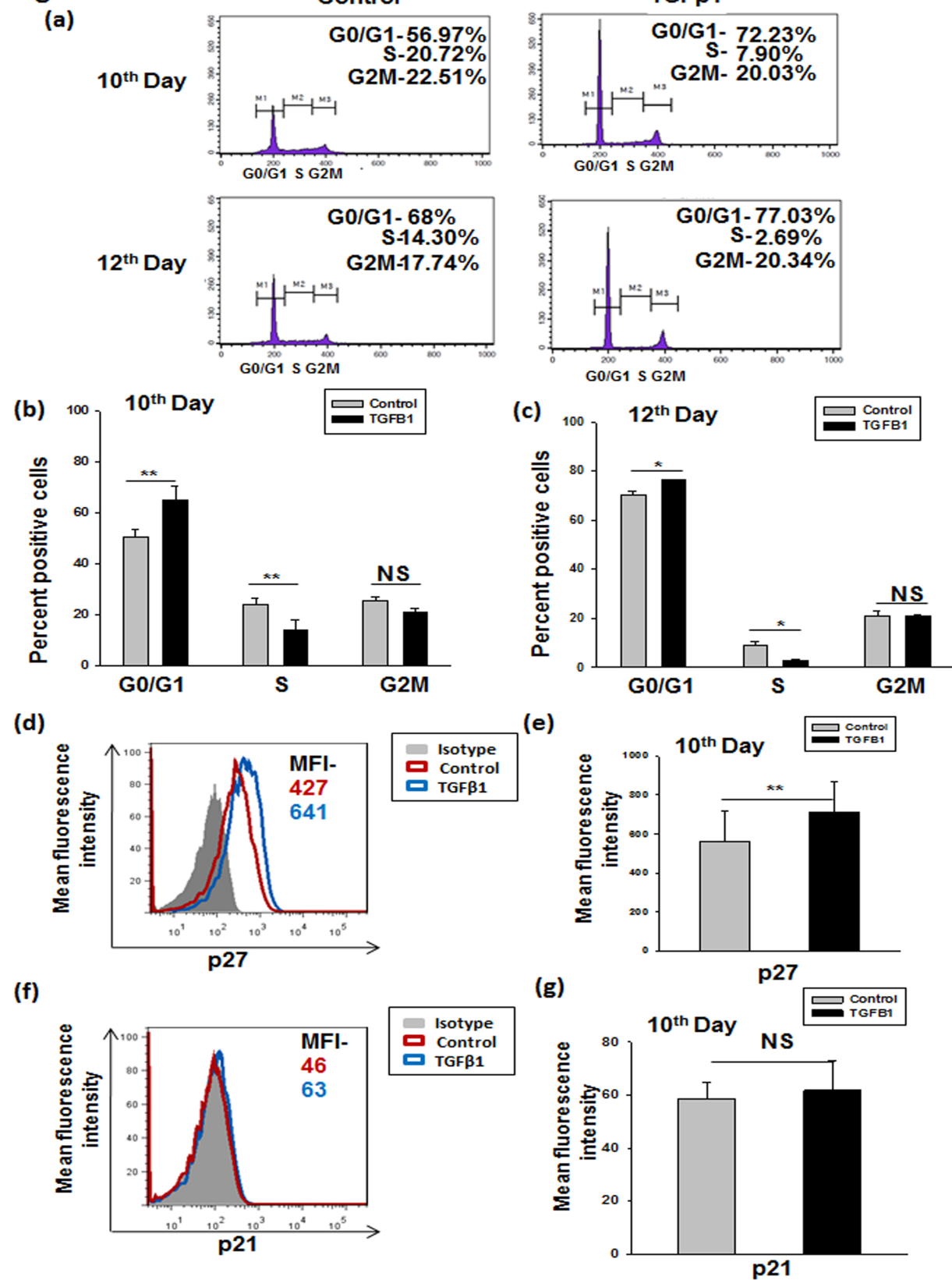

Supplement: Supplementary file 4 — Figure S4. TGF-β1 causes cell cycle arrest during erythropoiesis process. a) Representative flow cytometry overlay showing cell cycle profile of control and TGF-β1 set on day 10 and day 12. TGF-β1 supplementation significantly increases the G0/G1 phase and decreases the S phase on b) day 10 and c) day 12. Data show mean ± SEM from independent experiments done with cells from four different donors. *p < 0.05; **p < 0.01; NS=Non significant. d) Representative flow panel showing mean fluorescence intensity of p27. e) Graph shows an increase in mean fluorescence intensity of p27 in TGF-β1 set compared to the control set on day 10. Results are presented as mean ± SEM from independent experiments with four different donor samples **p < 0.01. f) Representative flow panel showing mean fluorescence intensity of p21. g) The graph shows TGF-β1 supplementation does not affect mean fluorescence intensity of p21 on day 10. Data show mean ± SEM from independent experiments done with cells from four different donors NS=Non significant. [file 13287_2020_1603_MOESM4_ESM.pdf]

Figure S5

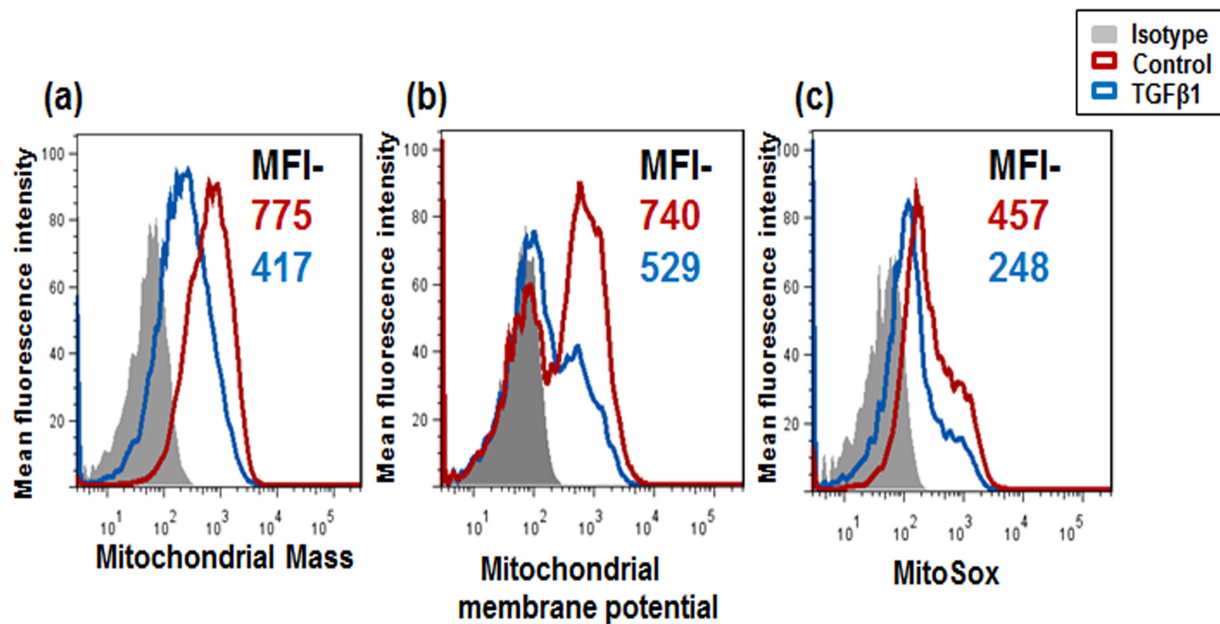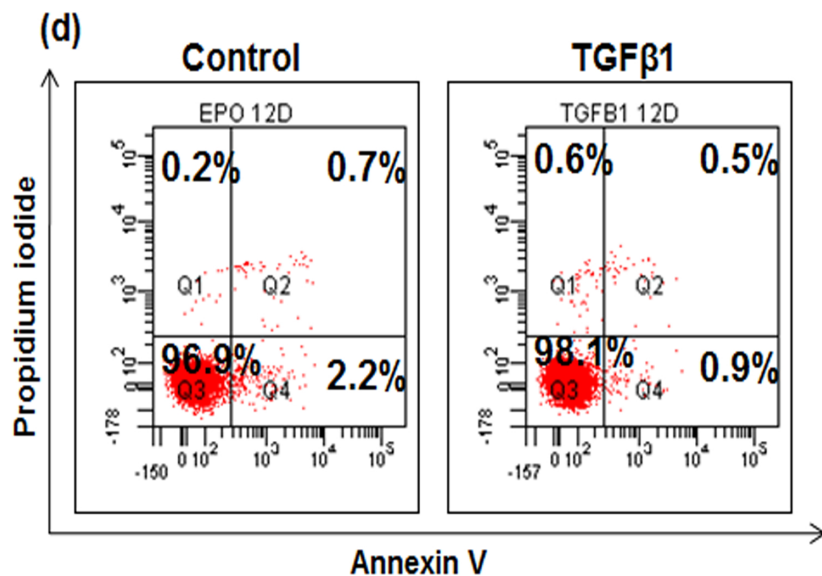

Supplement: Supplementary file 5 — Figure S5. TGF-β1 induces mitophagy in cultured cells. Representative overlays showing a decrease in a) Mitochondrial mass b) Mitochondrial membrane potential and c) Mitochondrial ROS in TGF-β1 set as compared to the control set. MFI: Mean fluorescence intensity. d) Representative dot plot showing apoptosis level of day 12 cultured cells. [file 13287_2020_1603_MOESM5_ESM.pdf]

**Figure S6**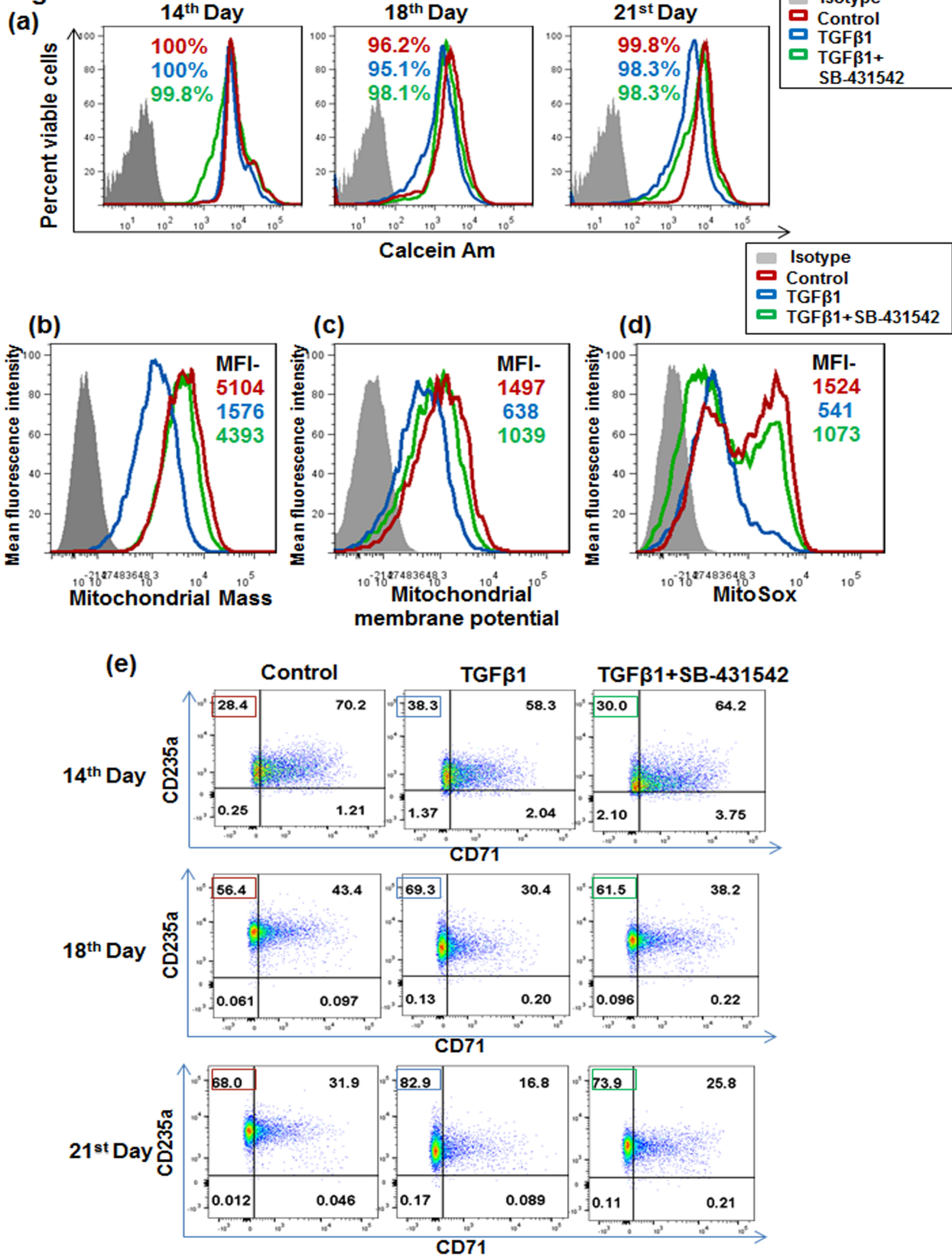

Supplement: Supplementary file 6 — Figure S6. TGF-β1 enhances RBC production by inducing mitophagy. Representative flow cytometry overlay showing a) Cells viability by Calcein Am staining b) Mitochondrial mass c) Mitochondrial membrane potential and d) Mitochondrial ROS after SB-431542 treatment. e) Dot plot showing a significant decrease in percent mature RBCs on days 14, 18 and 21 after SB-431542 treatment in TGF-β1 set. [file 13287_2020_1603_MOESM6_ESM.pdf]
